# N-terminal region of *Drosophila melanogaster* Argonaute2 forms amyloid-like aggregates

**DOI:** 10.1186/s12915-023-01569-3

**Published:** 2023-04-19

**Authors:** Haruka Narita, Tomohiro Shima, Ryo Iizuka, Sotaro Uemura

**Affiliations:** Department of Biological Sciences, Graduate School of Science, The University of Tokyo, Tokyo, Japan

**Keywords:** Argonaute, Aggregation, Amyloid, Prion, RNA interference

## Abstract

**Background:**

Argonaute proteins play a central role in RNA silencing by forming protein-small RNA complexes responsible for the silencing process. While most Argonaute proteins have a short N-terminal region, Argonaute2 in *Drosophila melanogaster* (DmAgo2) harbors a long and unique N-terminal region. Previous in vitro biochemical studies have shown that the loss of this region does not impair the RNA silencing activity of the complex. However, an N-terminal mutant of *Drosophila melanogaster* has demonstrated abnormal RNA silencing activity. To explore the causes of this discrepancy between in vitro and in vivo studies, we investigated the biophysical properties of the region. The N-terminal region is highly rich in glutamine and glycine residues, which is a well-known property for prion-like domains, a subclass of amyloid-forming peptides. Therefore, the possibility of the N-terminal region functioning as an amyloid was tested.

**Results:**

Our in silico and biochemical assays demonstrated that the N-terminal region exhibits amyloid-specific properties. The region indeed formed aggregates that were not dissociated even in the presence of sodium dodecyl sulfate. Also, the aggregates enhanced the fluorescence intensity of thioflavin-T, an amyloid detection reagent. The kinetics of the aggregation followed that of typical amyloid formation exhibiting self-propagating activity. Furthermore, we directly visualized the aggregation process of the N-terminal region under fluorescence microscopy and found that the aggregations took fractal or fibril shapes. Together, the results indicate that the N-terminal region can form amyloid-like aggregates.

**Conclusions:**

Many other amyloid-forming peptides have been reported to modulate the function of proteins through their aggregation. Therefore, our findings raise the possibility that aggregation of the N-terminal region regulates the RNA silencing activity of DmAgo2.

**Supplementary Information:**

The online version contains supplementary material available at 10.1186/s12915-023-01569-3.

## Background

RNA silencing is involved in various biological processes including antiviral defense, development, and the maintenance of genomic integrity [1]. Among the RNA silencing machinery, Argonaute proteins play key roles by directly binding to small RNAs to form the RNA-induced silencing complex (RISC) [2]. After the small RNA in RISC recognizes its RNA targets, Argonaute proteins display endonucleolytic activity or translational repression [3]. Despite the functional variety of Argonaute proteins, their structural organization is highly conserved, sharing four distinct domains: N, PAZ, MID, and PIWI [4]. Previous studies have revealed the functions of each domain. The N domain initiates duplex unwinding immediately after small RNA binds to Argonaute [5]. The PAZ and MID domains recognize the 5′ and 3′ ends of the small RNA, respectively [6, 7, 8]. The PIWI domain, which shares structural similarities with ribonucleases, cleaves the target RNA [9, 10].

In contrast to these highly conserved domains, the N-terminal region, which is located upstream of the N domain, shows extensive diversity among species [11]. While most Argonaute proteins have a short N-terminal region (e.g., 24 residues in human Ago2), Argonaute2 in *Drosophila melanogaster* (DmAgo2) harbors a long and unique N-terminal region. The N-terminal region (residues 1–398; henceforth, Nter) comprises almost one-third of the residues in DmAgo2 (1208 residues). Partial truncation of Nter (residues 326–371 deletion) has been reported to impair RNA silencing in mutant flies [12]. However, an in vitro study demonstrated that another DmAgo2 mutant with Nter deletion (residues 1–278 deletion) still forms RISC and retains RNA cleavage activity [13].

Many eukaryotic RNA-binding proteins contain a prion-like domain (PrLD), which is a subclass of amyloid-forming peptides and organizes intracellular condensates and regulates biochemical reactions [14, 15, 16, 17]. PrLDs consist of intrinsically disordered, low-complexity sequences often enriched in glutamine and glycine residues [18, 19]. The Nter sequence shares this property. The content of glutamine and glycine residues in DmAgo2 Nter reach approximately 40% and 20%, respectively [11, 12]. Inspired by these findings, here, we tested whether DmAgo2 Nter can form amyloid, which is a well-known structure of PrLD aggregates. Our in silico prediction and biochemical assays demonstrated that Nter can form amyloid. We also directly visualized by fluorescence microscopy how Nter aggregates and found that the aggregates are polymorphic. Since many amyloids have been shown to regulate biochemical reactions through their aggregate formation [14, 15, 16, 17], our results raise the possibility that Nter regulates RNA silencing activities in cells by forming aggregates.

## Results

### PrLD prediction based on the amino acid sequence of DmAgo2

**Fig. 1:**
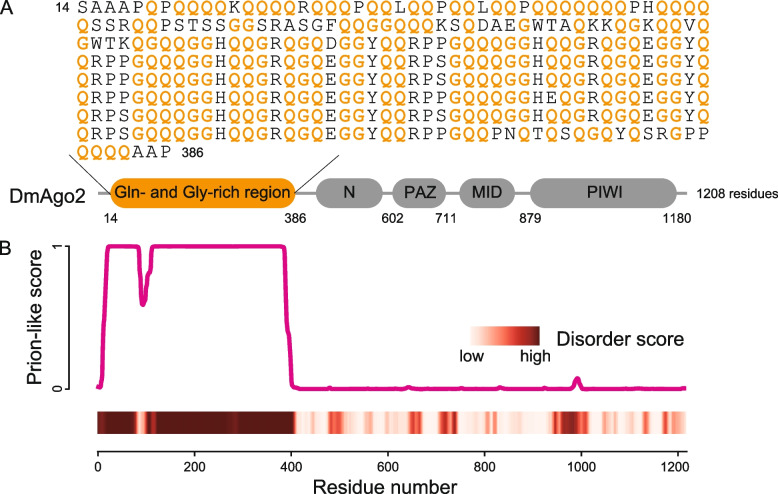
DmAgo2 has a long characteristic N-terminal region. **A** A schematic diagram of the full-length DmAgo2 protein and the sequence of N-ter. The four conserved domains are shown in gray, while the N-terminal region is shown in orange. In the N-ter sequence, glutamine and glycine residues are shown in orange. **B** Plot of the prion-like probability of the DmAgo2 sequence predicted by PLAAC (top) and a heatmap of the degree of disorder predicted by PONDR analysis (bottom)

To confirm the characteristics of DmAgo2 based on its amino acid sequence (Fig. 1A), we performed intrinsically disordered regions (IDRs) prediction and three kinds of PrLDs prediction. First, to confirm that DmAgo2 harbors IDRs, we analyzed the DmAgo2 amino acid sequence using PONDR [20], a neural network-based prediction algorithm of IDRs. Two parts of Nter (residues 1−87 and 105−412) were identified as regions with high disorder scores (Fig. 1B). Alphafold2 also predicted that Nter does not take a specific structure, supporting the notion that Nter is IDR [21]. Then, the possibility that Nter is not only an IDR but also a PrLD that can form amyloid was predicted by three PrLD prediction algorithms: the prion-like amino acid composition (PLAAC) [22], prion aggregation prediction algorithm (PAPA) [23], and PrionW [24]. PLAAC and PAPA predict PrLDs based on amino acid compositions. PLAAC, a hidden Markov model (HMM)-based prion prediction algorithm, uses the local context in the amino acid sequence, while PAPA only uses amino acid compositions [25]. On the other hand, PrionW utlizes amyloidogenicity in addition to amino acid compositions. The predictions of Nter being PrLD varied across the algorithms; PLAAC identified residues 14 − 386 in Nter as the region with a high prion-like score (Fig. 1B) while PAPA (Additional file 1: Fig. S1) did not. The PrionW algorithm provides its highest accuracy for yeast PrLD prediction when the pWALTZ cutoff value was set to 73.55. With this cutoff value, PrionW did not predict that DmAgo2 harbors PrLD. However, it has been reported that the algorithm requires lower cutoff values to correctly predict some well-characterized PrLDs in other organisms [24]. Nter was predicted as PrLD by PrionW when the cutoff value was set to be lower than 62.8, hinting at the possibility that Nter is PrLD. Because of the variation in the predicted results, we next experimentally tested whether Nter has the capacity to form amyloid.

### Nter can form SDS-resistant amyloid in a typical amyloid formation manner

**Fig. 2:**
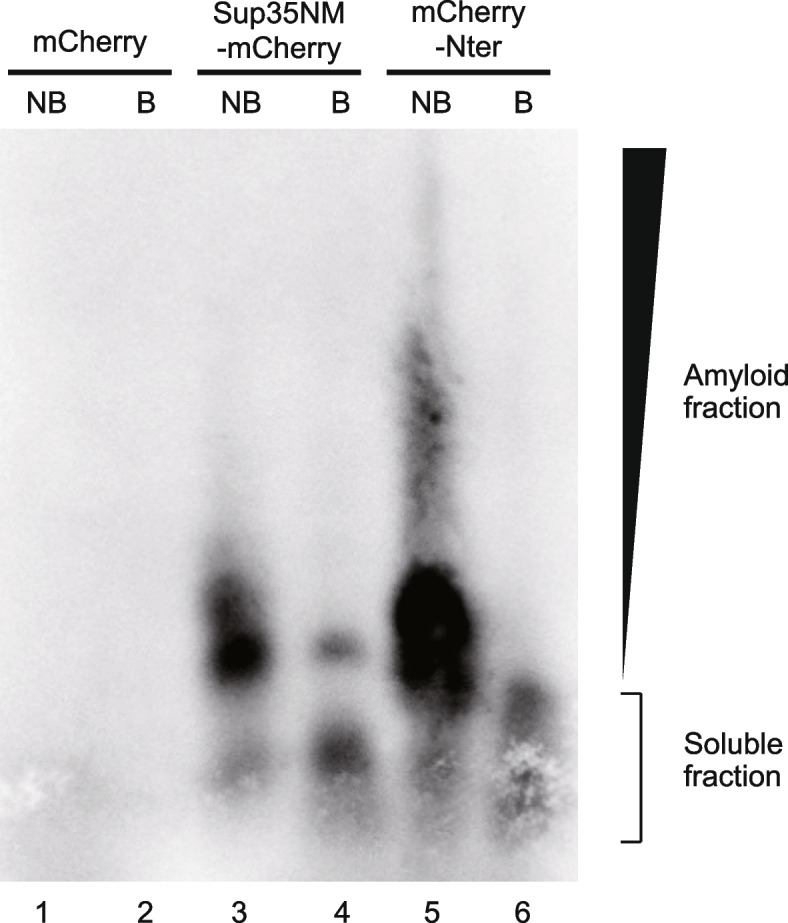
mCherry-Nter formed SDS-resistant aggregates. SDD-AGE analysis of mCherry-Nter aggregates. mCherry and Sup35NM-mCherry were used as reference controls. mCherry-tagged polypeptides were detected by western blotting with anti-RFP polyclonal antibodies. NB, non-boiled fraction; B, boiled fraction

We prepared a recombinant protein of Nter fused with mCherry at its N-terminal (mCherry-Nter) to test whether Nter behaves as a PrLD (Additional file 1: Fig. S2 [26], Additional file 2: Uncropped Image 1 and Uncropped Image 2). PrLDs are known to form aggregates, which are not disassembled even in the presence of sodium dodecyl sulfate (SDS) [27]. To test if Nter forms SDS-resistant aggregates, we first incubated 5 μM of monomeric mCherry-Nter in aggregation buffer (20 mM sodium phosphate, 50 mM sodium chloride, pH 7.4) for 3 days at room temperature. Then, the aggregates in the solution were collected by ultracentrifugation and subjected to semi-denaturing detergent agarose gel electrophoresis (SDD-AGE) [27]. As references for the assay, mCherry and Sup35NM-mCherry were also subjected to SDD-AGE. mCherry is a monomeric protein [28], whereas Sup35NM is a well-known PrLD and can form amyloid [29]. mCherry did not appear as a distinct band, indicating that mCherry did not form any aggregates (Fig. 2). Contrarily, Sup35NM-mCherry and mCherry-Nter predominantly migrated as smears with high molecular weights, suggesting that mCherry-Nter formed SDS-resistant aggregates similar to Sup35NM-mCherry (Fig. 2, Additional file 2: Uncropped Image 3). The aggregates of Sup35NM-mCherry and mCherry-Nter were dissolved into soluble fractions when boiled. Thus, the results showed that Nter forms SDS-resistant aggregates.

**Fig. 3:**
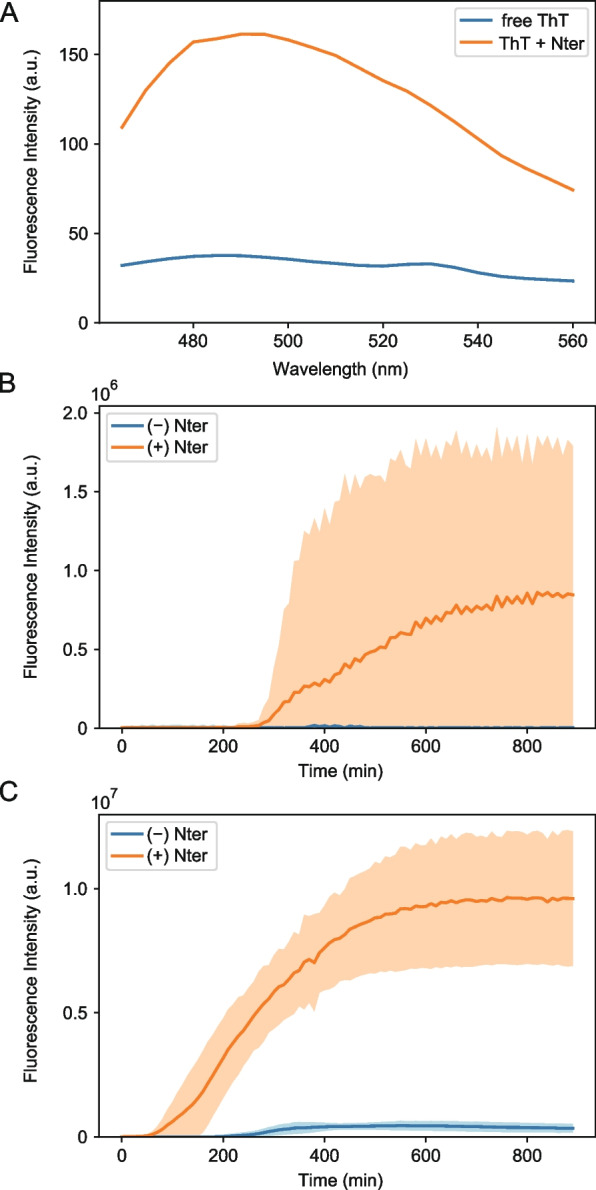
Nter aggregated in the same manner as common amyloids. **A** Fluorescence spectra of ThT in the absence (blue) and presence (orange) of Nter aggregates. **B** Time course of the ThT fluorescence intensity in the absence (blue) and presence (orange) of mCherry-Nter monomers. The solid curves and filled area indicate the mean value and standard deviation of three repetitive experiments (*n* = 3), respectively. **C** Time course of ThT fluorescence intensity in the absence (blue) and presence (orange) of mCherry-Nter seeds. The solid curves and filled area indicate the mean value and standard deviation of three repetitive experiments (*n* = 3), respectively

To further test whether Nter aggregates form amyloid, we performed thioflavin-T (ThT) assays. ThT is a fluorescent probe with specific fluorescence enhancement upon binding with amyloid fibrils [30, 31]. First, we measured the fluorescence spectrum of 20 μM ThT with and without 5 μM Nter solution incubated for 12 h beforehand. In the presence of Nter, the fluorescence intensity of ThT was enhanced approximately four times compared to that without Nter, and a peak at 490 nm was clearly visible in the fluorescence spectrum (Fig. 3A), indicating the presence of amyloid in the Nter solution.

To confirm that these aggregates were formed in a typical amyloid formation manner, we tracked the aggregation process over time by monitoring the ThT fluorescence intensity under microscopy. Consistent with the canonical amyloid formation from monomers generally requiring a lag phase for nucleation [32], the ThT fluorescence intensity with Nter showed an initial lag phase of ~ 300 min and then reached a plateau at ~ 800 min after mixing monomeric Nter with ThT in aggregation buffer (Fig. 3B, Additional file 1: Fig. S3A, Additional file 3: Table S1). Prion-like proteins generally exhibit seeded self-propagation in vivo and in vitro [29, 33]. Therefore, we prepared the seeds of Nter aggregates and examined whether the seeds can accelerate the aggregation process. The addition of the seeds reduced the initial lag phase to less than 100 min (Fig. 3C, Additional file 1: Fig. S3B, Additional file 4: Table S2), showing that the Nter aggregation process follows canonical amyloid formation kinetics with a self-propagation property.

On the other hand, Nter also exhibited properties uncommon for canonical amyloid. The ThT fluorescence intensity outside of the visible Nter aggregates showed the highest peak immediately after mixing ThT with monomeric Nter (Additional file 1: Fig. S3). This unusual property was also observed in ThT fluorescence measurements using a plate reader (Additional file 1: Fig. S4, Additional file 5: Table S3). As ThT recognizes β-sheet structure in amyloid, the results raise the possibility that transient β-sheet structure in Nter monomer or oligomer may unwind over time during the aggregation process.

### Direct visualization of Nter aggregates

**Fig. 4:**
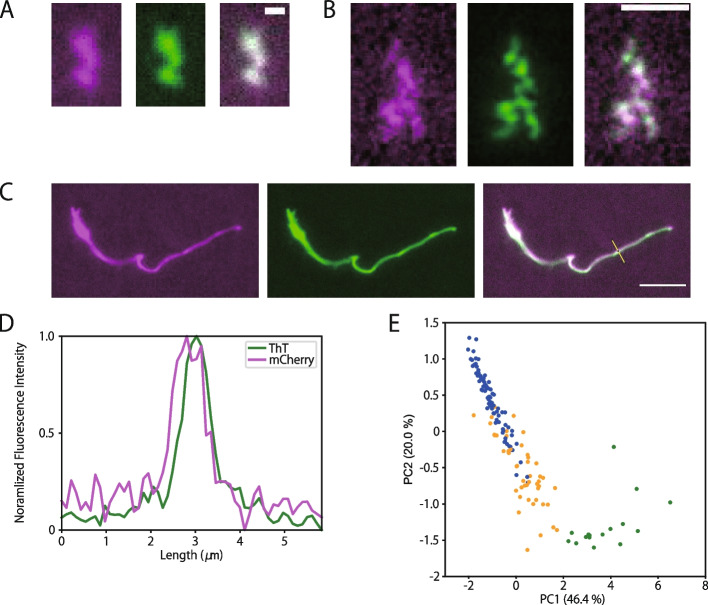
Fluorescence imaging of the Nter aggregate morphology. **A**–**C** Representative fluorescence images of linear fractal-shaped (**A**), branched fractal-shaped (**B**), and fibril-shaped aggregates (**C**). Fluorescence signals from mCherry (left panels, magenta) and ThT (middle panels, green) showed almost identical images. Merged images are shown in the right panels. Scale bars represent 1 μm (**A**, **B**) and 5 μm (**C**). **D** Fluorescence intensity profiles of mCherry and ThT along the line in the merged image of (**C**). Intensities were normalized. **E** PCA analysis of the shapes of the Nter aggregates. The plots were colored according to the classified group (blue, linear fractal-shaped; green, branched fractal-shaped; orange, fibril-shaped)

Next, we directly visualized the shape of Nter aggregates using fluorescence microscopy to investigate their morphology. The fluorescence of mCherry-Nter that had been flushed into a glass chamber and incubated for 12 h to form aggregates revealed fractal or fibril-shaped aggregates. When ThT was added to the chamber, the fluorescent images of ThT and mCherry matched completely, suggesting that the entire region of the Nter aggregates took an amyloid structure (Fig. 4A − D).

Since the aggregates showed various shapes (Fig. 4A–C and Additional file 1: Fig. S5), we analyzed and classified the shapes using principal component analysis (PCA) with *k*-means clustering into three groups: linear fractal-shaped, branched fractal-shaped, and fibril-shaped aggregates (Fig. 4E). The most frequently observed group (56%) was linear fractal-shaped aggregates (Fig. 4A and Additional file 1: Fig. S5A). In these aggregates, ~ 0.5-μm fluorescent puncta were linearly connected to each other. The median length of these aggregates was 1.4 [1.2–2.1] μm (median with quartile range, *n* = 83, Additional file 1: Fig. S6A), corresponding to ~ 2 puncta. For the second-largest group, 32% of the whole aggregates were classified as branched fractal-shaped aggregates (Fig. 4B and Additional file 1: Fig. S5B). In the aggregates of this group, several puncta were bound to branch off from the main chain of the aggregates. The median length of the longest chain of the aggregates was 3.3 [2.2–4.9] μm (median with quartile range, *n* = 47, Additional file 1: Fig. S6B), and the median number of branched points was 4 [3–6] (median with quartile range, *n* = 47, Additional file 1: Fig. S6C). The remaining 12% of the whole aggregates were classified as fibril-shaped aggregates (Fig. 4C and Additional file 1: Fig. S5C). This fibril shape is the most common structure for amyloids [34]. The median length of the Nter fibril was 27 [19–50] μm (median with quartile range, *n* = 17, Additional file 1: Fig. S6D), taking a shape much longer than the fractal-shaped aggregates. Most Nter fibrils contained a few numbers of puncta, but these puncta did not directly bind to each other. Thus, we could classify the shapes of the Nter aggregates according to the presence of branching, the length of the aggregates, and the alignment of puncta.

**Fig. 5:**
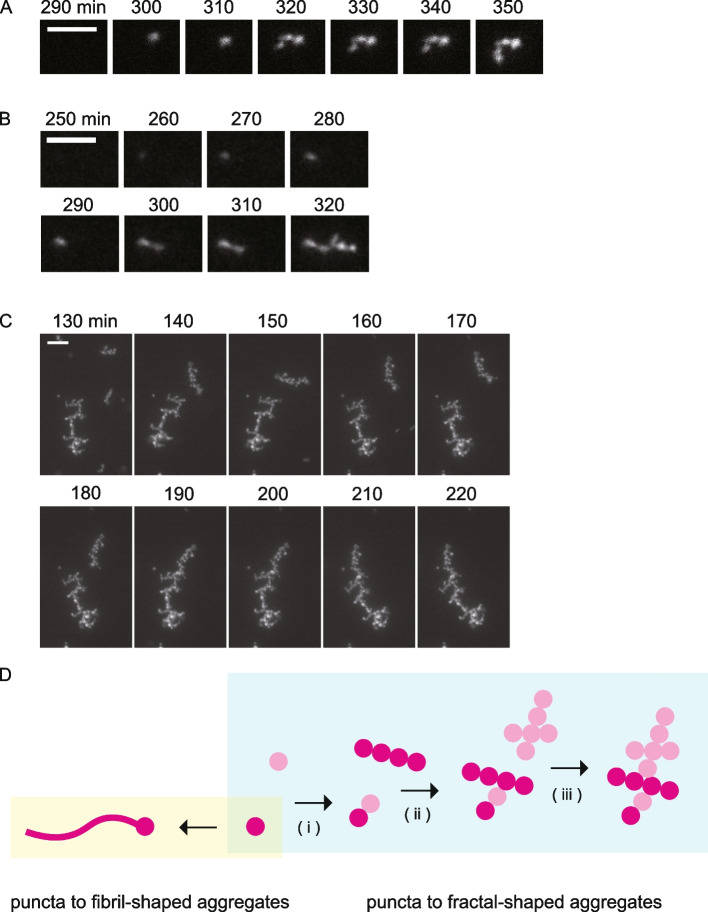
Fractal-shaped Nter aggregates grew by docking between puncta. **A–C** Time-lapse images of the Nter aggregate formation. Ten-minute intervals are shown from left to right. Scale bars represent 5 μm (**A**,** B**) and 10 μm (**C**). **D** Model of the aggregate formation. Assuming both fibril- and fractal-shaped Nter aggregates grew from a single punctum, the polymerization of Nter monomers from the punctum results in fibril-shaped aggregates, while the fractal-shaped aggregates result from puncta binding to each other (i). The binding of several linear fractal-shaped aggregates produces branched fractal-shaped aggregates (ii). The branched fractal-shaped aggregate becomes bigger by docking with other fractal-shaped aggregates (iii)

Time-lapse imaging of the Nter aggregation process demonstrated how the fractal-shaped aggregates formed. First, fluorescent puncta docked to each other, forming linear fractal-shaped aggregates (Fig. 5A). Next, linear fractal-shaped aggregates docked to each other, forming branched fractal-shaped aggregates (Fig. 5B). Finally, branched fractal-shaped aggregates docked to each other, forming larger branched fractal-shaped aggregates (Fig. 5C). Therefore, it is most likely that the linear and branched fractal-shaped aggregates share the same formation process, and the difference in the stage of the formation process is seen as the difference in shape.

To obtain higher-resolution images of the shapes of the aggregates, we also performed transmission electron microscopy (TEM) analyses. The TEM images of Nter aggregates demonstrated the presence of both fractal- and fibril-shaped aggregates as shown by fluorescence microscopy (Additional file 1: Fig. S7). In the fractal-shaped aggregates, ~ 0.5-mm puncta, which were also observed by fluorescence microscopy, had smaller fractal shapes consisting of smaller puncta of approximately 20 nm in diameter (Additional file 1: Fig. S7A and B). This result suggests that puncta aggregates are formed by the docking of smaller puncta. Fibril-shaped aggregates of Nter had a large width (Additional file 1: Fig. S7C and D), unlike common amyloid fibrils, which are only 10–20 nm in diameter [30]. The median widths of the fibril-shaped aggregates shown in Additional file 1: Fig. S7C and S7D were 150 [130–170] nm and 1.2 [0.9–1.4] μm (median with interquartile range, *n* = 947 pixels, Additional file 1: Fig. S8A; *n* = 2346 pixels, Additional file 1: Fig. S8B). The width was not uniform over the entire length of the aggregates, and some Nter fibrils were observed to have a twisted structure (Additional file 1: Fig. S7D). Taken together, the TEM images showed results that supported the fluorescence microscopy data, revealing finer structures for both fractal- and fibril-shaped aggregates.

## Discussion

Here, we demonstrated that Nter can form amyloid-like aggregates by in silico prediction and biochemical assays. The PLAAC algorithm predicted Nter is a PrLD that can form prion-like aggregates, a subclass of amyloids, based on its characteristic sequence. mCherry-Nter fusion protein indeed formed aggregates that exhibited amyloid-specific properties of resistance to SDS and binding to ThT. Furthermore, Nter aggregated in a manner consistent with typical amyloids, including a lag phase for nucleation and self-propagation activity. These results strongly suggest that Nter can act as an amyloid-forming peptide.

We found that Nter aggregates can take two distinct conformational states. Most aggregates took fractal shapes, while the remaining had fibril shapes. This is in contrast to canonical amyloids that form into fibrils; therefore, we classified the Nter aggregates as amyloid-like, not amyloid. However, the existence of the fractal-shaped aggregates is consistent with previous studies on self-assembled proteins showing that fractal-shaped aggregates tend to form under diffusion-limited conditions [35, 36, 37, 38], because protein diffusion is limited in the 145-μm-thick chamber in our assays. Yet, it remains to be elucidated whether these observed fractal-shaped aggregates are actually formed in cells. During our observation, fractal-shaped aggregates docked to form larger fractal-shaped aggregates. On the other hand, fibril-shaped aggregates frequently contained a few numbers of puncta in their fibrils (Fig. 4C and Additional file 1: Fig. S5C). Although the forming process of fibril-shaped aggregates was not observed, this finding implies that fibril-shaped aggregates can elongate from the edges of the puncta. Taken together, we propose a growth model of Nter aggregates in which the docking of several puncta forms fractal-shaped aggregates and polymerization of monomeric Nter from a single punctum forms fibril-shaped aggregates (Fig. 5D).

Another unusual property of Nter as canonical amyloid is that its monomer as well as the aggregates showed a high ThT fluorescence signal (Additional file 1: Figs. S3, S4). Because ThT recognizes β-sheet-rich structures in amyloid, the result suggests the presence of a transient β-sheet structure in the Nter monomers. The transient structure could be derived from two types of polyglutamine sequences in Nter: Q_7_PHQ_5_ and repeats of Q_3_GGHQ_2_. Polyglutamine repeats have been reported to take intermediate structures including intramolecular β-sheets [39, 40, 41]. Therefore, there is a possibility that the polyglutamine sequences in the Nter monomer transiently fold into β-sheet structures and bind with ThT. If so, the transient β-sheets would facilitate the aggregation process through intermolecular interactions [40], while kinks of the histidine and proline residues in the polyglutamine sequence could increase the solubility of the monomer and have an inhibitory effect on aggregation [39]. In contrast to the monomeric case, the Nter aggregation process showed a similar trend of increase in the ThT fluorescence signal as canonical amyloids, suggesting that the Nter aggregates contain abundant β-sheet structures similar to common amyloids.

We focused on DmAgo2 because *Drosophila melanogaster* is one of the most well-studied model organisms in RNA silencing studies. However, the aggregation of the N-terminal region may be a general phenomenon not limited to DmAgo2. Although Argonaute proteins of many organisms have only a short N-terminal region, among arthropods, Ago2 proteins share glutamine- and glycine-enriched properties in the N-terminal region [42]. Their amino acid composition, which is a major factor in amyloid-formation potential [43], is close to that of DmAgo2 Nter. Therefore, the N-terminal regions of Ago2 in other arthropods may also form amyloid-like aggregates. Consistent with these notions, a comprehensive phylogeny-wide PrLD prediction in the Argonaute protein family suggested that the proteins in many species including arthropods and plants conserve the N-terminal PrLD (Additional file 1: Fig. S9). Among them, we confirmed that the N-terminus of Ago1 in *Arabidopsis thaliana* that possesses a glutamine- and glycine-rich region indeed formed amyloid-like aggregates similar to DmAgo2 Nter (Additional file 1: Fig. S10). The physiological features and function of the aggregation property of the N-terminal regions await further study, but the property has the potential to function as a regulatory mechanism for RNA silencing across species.

## Conclusions

Here, we found a new property of the N-terminal region of DmAgo2. Nter aggregation may indirectly modulate the RNA silencing activity of DmAgo2. It has been shown that Nter is not essential for DmAgo2 to exhibit RNA silencing activity under in vitro conditions, but the partial truncation of Nter causes RNA silencing defects in the compound eyes of flies [12]. Combining these previous studies with our current results raises the possibility that in vivo Nter aggregation modulates RISC formation and/or RNA cleavage activity by other domains of DmAgo2. Protein aggregation can both be inhibitory on protein activity by steric hindrance effects on the substrate binding or facilitative by increasing local protein concentrations [44]. Furthermore, it has been reported that protein aggregation is a major driving force for liquid–liquid phase separation in the cytoplasm, allowing for the proper subcellular localization of biomolecules [14]. Indeed, DmAgo2 is localized in cytoplasmic D2 bodies, where endogenous siRNAs are loaded onto DmAgo2, and this localization is essential for the formation of RISC with the proper small RNA [45]. The ability of Nter to form aggregates may contribute to such modulation of DmAgo2 activity and localization. Considering the sizes of cytoplasm and the aggregates, these functional aggregates are likely limited to puncta-shaped aggregates or oligomers. Although it still needs to be verified whether the aggregation of endogenous DmAgo2 actually occurs in cells, this hypothesis can explain the apparent inconsistency among previous in vitro and in vivo studies regarding the effects of Nter in RNA silencing.

## Methods

### In silico analysis

The amino acid sequence of DmAgo2 (equivalent to UniProt entry Q9VUQ5 with the deletion of residues 43–48) was derived from native *Drosophila* DNA. The sequence was analyzed using the programs PLAAC (http://plaac.wi.mit.edu/) [22], PAPA (https://combi.cs.colostate.edu/supplements/papa/) [46], and PrionW (http://bioinf.uab.cat/prionw/) [24] with default settings. Disorder scores were calculated using the Predictor of Natural Disordered Regions (PONDR) algorithm with the VLXT predictor (http://www.pondr.com/) [47].

The following analysis was carried out using Python. The amino acid sequence of full-length Argonaute family protein, including isoforms, was obtained from the UniProt database. The presence of PrLDs was analyzed using PLAAC. The organisms from which the Argonaute family proteins were derived were classified at the phylum level based on the NCBI database (https://ftp.ncbi.nlm.nih.gov/pub/taxonomy/taxdump.tar.gz). The results were plotted using a Python plotting library (Matplotlib: http://matplotlib.org).

### Plasmid construction

The expression plasmid for N-terminal 6 × His-tagged mCherry-Nter was constructed by inserting the sequence encoding DmAgo2 Nter (residues 1–398) amplified from the native DmAgo2 sequence into pCold I (TAKARA Bio) using the InFusion HD cloning kit (Clontech). The expression plasmid for C-terminal 6 × His-tagged Sup35NM-mCherry was constructed by inserting the sequence encoding mCherry into the expression plasmid for Sup35NM based on pET29b (residues 1–253) (a kind gift from Prof. M. Tanaka (RIKEN CBS, Japan)).

### Protein expression and purification

For the protein expression, *E. coli* BL21(DE3) cells were transformed with the constructed plasmids and selected using 50 mg/L carbenicillin (for pCold I and pET11a plasmids) or 50 mg/L kanamycin (for pET29b plasmid). These antibiotics were added to all culture media described below. The transformed *E. coli* cells were pre-cultured in 5 mL of LB medium overnight at 37 °C and then inoculated into 2 L of TB medium. The cells were grown at 37 °C until OD_600_ reached 1.2 (6 × His-tagged mCherry-Nter and 6 × His-tagged Sup35NM-mCherry) or 0.6 (mCherry-His). 6 × His-tagged mCherry-Nter was expressed with 1 mM IPTG at 12 °C. mCherry-His and 6 × His-tagged Sup35NM-mCherry were expressed with 1 mM IPTG at 28 °C. After 20 h of culture, the cells were harvested and then lysed in 80 mL of lysis buffer (20 mM Hepes–KOH pH8.0, 100 mM NaCl, and 6 M GdnHCl) with 10-s sonication at an intensity of 2 on ice using the ultrasonic disruptor (UD211, TOMY) a total of 9 times with 20-s intervals in between. Following centrifugation of the lysate at 18,000 × *g* for 30 min, 6 mL of 2 × Ni–NTA resin (Ni–NTA Agarose HP, FUJIFILM Wako Pure Chemical) was added to the supernatant. After a 60-min rotation at room temperature, the solution was transferred to a disposal column (Muromac Mini-column M, Muromachi Chemical Inc.). The resin was washed twice with 3 column volumes (CVs) of wash buffer (40 mM imidazole–HCl, 20 mM Hepes–KOH, pH8.0, 100 mM NaCl, and 6 M GdnHCl) and eluted with 1 CV of elution buffer (400 mM imidazole–HCl, 20 mM Hepes–KOH, pH8.0, 100 mM NaCl, and 6 M GdnHCl) three times. The elution fractions were resolved on 12% denaturing polyacrylamide gels and visualized by Coomassie Brilliant Blue (Nacalai Tesque) staining. Elution fractions containing proteins of interest were pooled and ultrafiltered to remove large aggregates using Vivaspin 6, 100,000 MWCO (Sartorius Stedim Biotech GmbH). Finally, the flow-through was concentrated using Vivaspin 6, 30,000 MWCO (Sartorius Stedim Biotech GmbH). The purified proteins were snap-frozen in liquid nitrogen and stored at −80 °C until use.

Monomers of mCherry-Nter, mCherry, and Sup35NM-mCherry were prepared as follows. First, purified proteins were diluted 50 times with aggregation buffer (20 mM sodium phosphate, pH 7.4, 50 mM NaCl). Next, the protein solution was ultracentrifuged at 418,000 × *g* for 20 min at 25 °C to remove large aggregates. Following the ultracentrifugation, the supernatant, equivalent to a half volume of the solution, was immediately transferred to a new 1.5-mL tube. The protein concentrations were determined using the following molar extinction coefficients at 280 nm: mCherry-Nter, 66,240 M^−1^ cm^−1^; Sup35NM-mCherry, 65,670 M^−1^ cm^−1^; and mCherry, 35,870 M^−1^ cm^−1^. The molar extinction coefficients were calculated using the ProtParam tool of ExPASy (https://web.expasy.org/protparam/) [48]. Then, the protein solution was diluted to 5 μM with aggregation buffer. Nter aggregates were prepared by incubating the above solution at room temperature for the given time.

Seeds were prepared as follows. First, purified mCherry-Nter was diluted 10 times with elution buffer. Next, the solution was applied to a gel filtration spin column (Micro Bio-Spin 30 column, Bio-Rad) equilibrated with aggregation buffer to remove GdnHCl and ultracentrifuged at 418,000 × *g* for 20 min at 25 °C to remove large aggregates. Finally, the supernatant, equivalent to 90% solution volume, was immediately transferred to a new 1.5-mL tube as a seed.

### SDD-AGE

After monomers were incubated for 3 days at room temperature without agitation, aggregates were pelleted by ultracentrifugation at 418,000 × *g* for 20 min at 25 °C. After completely removing the supernatant, the pellet was resuspended in an aggregation buffer. Prepared samples were subjected to SDD-AGE analysis. First, aggregate solutions were mixed with 4 × sample buffer (2 × TAE, 20% (v/v) glycerol, 4% (w/v) SDS, 0.25% (w/v) bromophenol blue). Next, after incubation for 15 min at room temperature with or without heat treatment at 95 °C for 2 min, the samples were loaded onto a 1.5% agarose gel containing 1 × TAE and 0.1% SDS. Finally, the aggregates and monomer protein were resolved on the gel in running buffer (1 × TAE, and 0.1% SDS) at 75 V for 90 min at 4 °C, followed by capillary blotting onto a PVDF membrane (FUJIFILM Wako Pure Chemical) for the western blotting analysis, as described previously [27]. Anti-RFP polyclonal antibody (1/5000 dilution from the product, PM005, MBL) and HRP-labeled IgG detector (1/5000 dilution from the product, Western BLoT Rapid Detect v2.0, Takara Bio) were used as primary and secondary antibodies, respectively. Proteins were detected with SuperSignal West Femto (Thermo Scientific) on a gel imager (Amersham Imager 600, Cytiva).

### Measurement of the spectrum

A total of 5 μM mCherry-Nter monomers were incubated for 12 h at 25 °C to prepare mCherry-Nter aggregates. Fluorescence spectra of mCherry-Nter aggregates in solution with or without 20 μM ThT (FUJIFILM Wako Pure Chemical) were obtained using a fluorescence spectrometer (RF-6000, Shimadzu) at room temperature. The excitation wavelength was set at 455 nm (bandwidth: 5 nm), and the emission was recorded from 465 to 560 nm (bandwidth: 5 nm).

### Time course measurements of ThT fluorescence intensity by a microplate reader

The fluorescence intensity of ThT was monitored using a microplate reader (Infinite 200Pro, TECAN). The fluorescence at the wavelength of 480 nm (bandwidth: 20 nm) was measured with excitation at 450 nm (bandwidth: 9 nm) and a gain of 100. The kinetics were measured for 24 h every 10 min at 25 °C. The blank intensity was subtracted from the values of the monomeric mCherry-Nter solution.

### Chamber preparation for fluorescence microscopy

Flow chambers were prepared as described previously [49] with some modifications. Briefly, the coverslips (No. 1S 22 × 22 mm and No. 1S 24 × 32 mm, Matsunami) were cleaned in 1 N KOH for 15 min with sonication (Bransonic tabletop cleaner, Emerson). All subsequent preparation procedures were performed in a clean hood (Matsusada Precision). After 20 times rinsing with Milli-Q water and drying in a dryer, the coverslips were cleaned using a plasma cleaner (YHS-R, SAKIGAKE-Semiconductor Co., Ltd.). A 25-μL volume micro-chamber was made by placing a small coverslip of 22 × 22 mm over a 24 × 32 mm glass coverslip using double-sided adhesive tape (145 μm thickness, TERAOKA SEISAKUSHO CO., LTD.) in a clean hood. First, Lipidure-BL103 (NOF Corporation) was flowed into the chamber to coat the glass surface. After a 2-min incubation and excess Lipidure-BL103 removal by three washes with 25 μL aggregation buffer, each sample was flushed into the glass chamber.

To evaluate the colocalization of ThT and mCherry and the size distribution of the aggregates, mCherry-Nter aggregates were prepared by a 12-h incubation of monomeric mCherry-Nter at room temperature. mCherry-Nter aggregates were observed as follows. The mCherry-Nter aggregate solution was flushed into a Lipidure-coated glass chamber and incubated for 2 min at room temperature. Finally, excess mCherry-Nter unbound on the glass surface was removed by three washes with 25 μL of aggregation buffer with 20 μM ThT.

To measure the aggregation kinetics with or without the aggregate seeds, 5 μM mCherry-Nter monomers were flushed into a Lipidure-coated glass chamber and sealed with VALAP (1:1:1 mixture of vaseline, lanolin, and paraffin). In the presence of seeds, the final 5 μM of seeds was added to the monomer solution.

### Fluorescence microscopy

mCherry-Nter aggregates were observed using an inverted microscope (Nikon Ti-E) equipped with a filter cube for mCherry imaging consisting of an excitation filter (#67–033, Edmund Optics), a dichroic mirror (#67–083, Edmund Optics), an emission filter (#67–036, Edmund Optics), and a filter cube for ThT imaging consisting of an excitation filter (#67–026, Edmund Optics), a dichroic mirror (#67–078, Edmund Optics) and an emission filter (#67–028, Edmund Optics). mCherry and ThT were illuminated with a 532-nm laser (OBIS LX/LS, Coherent) and 445-nm LED light (SOLIS-445C, THORLABS), respectively. The images were obtained through a Plan Fluor 10 × /0.30 or Plan Apoλ 40 × /0.95 objective (Nikon) and recorded at 10 frames/s using an Orca Flash4.0 V3 digital CMOS camera (Hamamatsu Photonics). All equipment in the microscopy system was controlled by the Micro-Manager software [50]. To measure aggregate formation kinetics, ThT fluorescence images were recorded at 10-min intervals.

### Electron microscopy

After incubation of mCherry-Nter monomer for 24 h at room temperature without agitation, the aggregates were pelleted by ultracentrifugation at 418,000 × *g* for 20 min at 25 °C. After the complete removal of the supernatant, the pellet was resuspended in an aggregation buffer. An aliquot of the solution was placed on carbon-coated copper grids (ELS-C10 STEM Cu100P, Okenshoji) and incubated for 3 min at room temperature, and the excess solution was removed with filter paper. The incubation process was repeated two more times to ensure that a sufficient number of mCherry-Nter aggregates were adsorbed on the grid. The grids were then stained with a 1% (w/v) uranyl acetate solution, and the excess liquid on the grids was removed with filter paper. Electron micrographs were acquired using a transmission electron microscope (JEM-1400Flash, JOEL) with an acceleration voltage of 100 kV. Images were acquired with a high-sensitivity scientific CMOS camera (EM-14661 Flash, JOEL) at 800, 6000, 10,000, and 12,000 magnifications.

### Image analysis

Image analysis was performed using either ImageJ or Python. To accurately evaluate the colocalization of ThT and mCherry, we corrected the chromatic aberration of the images obtained from both fluorescence channels using ImageJ and the TurboReg plugin (http://bigwww.epfl.ch/thevenaz/turboreg/). The translational distortion parameters were obtained by comparing the averaged image from 10 images of an objective micrometer (OB-M, 1/100, Olympus) in each channel. Four regions of interest (3 × 3 pixels) were used for the calculation to minimize artifacts. The images of mCherry-Nter aggregates corrected using the obtained parameters were used for further colocalization analysis.

To classify the shapes of the aggregates observed by the ThT fluorescence, we first extracted the following features: the longest chain length, the mean width, the mean width per longest chain length, the total fluorescence intensity, the number of branched points per longest chain length, the number of fluorescence intensity peaks per longest chain length, and the percentage of the longest chain length to total length. To calculate these features, the images were preprocessed as described below. First, the background of each image was subtracted using the “Subtract Background” algorithm in ImageJ with a rolling ball radius of 200 pixels. Next, bright particles with more than 9 pixels with fluorescence intensity beyond the given threshold (60 a.u. out of 65,535 a.u.) were detected as aggregates. Following the above preprocessing, to calculate the total length of each aggregate, the “Skeletonize” algorithm was applied to each detected aggregate. Next, the longest chain length and the number of branched points of each aggregate were calculated from the skeletonized images. The mean width of each aggregate along the longest chain was calculated by averaging the widths along the longest chain using ImageJ. The number of fluorescence intensity peaks per longest chain length was detected using the Laplacian of Gaussian (LoG) method available in the scikit-image module in Python [51]. This number was evaluated because the smallest unit of a fractal-shaped aggregate is a round-shaped aggregate detected as a fluorescence punctum. Finally, we performed a PCA analysis based on the extracted eight features to obtain the two axes corresponding to the maximum variation (PC1) and second-most variation (PC2). Using the plot, the *k*-means clustering algorithm classified these aggregates into four groups. One of these groups consisted of only one sample, where both PC1 and PC2 values were outliers because two ends of the aggregate were connected to form a ring. We re-classified this one sample into “fibril-shaped aggregate.” The PCA result was plotted using a Python plotting library (Matplotlib: http://matplotlib.org) [52].

To calculate the amount of ThT bound to the aggregates at each time point, bright particles with more than 9 pixels of high fluorescence intensity (more than 250 a.u. out of 65,535 a.u.) were detected as the aggregates. ThT fluorescence intensity of the pixels with the detected aggregates was plotted against time.

To evaluate the width of fibril-shaped aggregates acquired by TEM, the images were processed as described below. Initially, fibril-shaped aggregates (fibrils) were traced and straightened using the ImageJ tools Segmented Line and Straighten. The width of the fibril was calculated as the distance between two peaks of the intensity value of the line perpendicular to the fibril longitudinal direction. Intensity peaks were detected using a module of scipy.signal.find_peaks [53].

## Supplementary Information

**Additional file 1: Fig. S1.** PAPA did not predict Nter as PrLD. **Fig. S2.** Gel electrophoresis analyses of the purified mCherry-Nter monomer. **Fig. S3.** Time-lapse images of Nter aggregation in the glass chamber. **Fig. S4.** Time-course ThT fluorescence measurement of Nter in solution. **Fig. S5.** Representative images of Nter aggregates. **Fig. S6.** Characteristics of each type of Nter aggregate. **Fig. S7.** TEM images of Nter aggregates. **Fig. S8.** Width distribution of fibril-shaped aggregates. **Fig. S9.** Comprehensive PrLD prediction for Argonaute family proteins. **Fig. S10.** *Arabidopsis thaliana *Ago1 forms amyloid-like aggregates.

**Additional file 2: Uncropped Image 1.** Uncropped gel image used for Additional file 1: Fig. S2A (A). **Uncropped Image 2.** uncropped blot image used for Additional file 1: Fig. S2B (B). **Uncropped Image 3.** Uncropped blot image used for Fig. 2.

**Additional file 3: Table S1.** Mean fluorescence intensity of detected aggregates of three experimental replicates for Fig. 3B.

**Additional file 4: Table S2.** Mean fluorescence intensity of detected aggregates of three experimental replicates for Fig. 3C.

**Additional file 5: Table S3.** Mean fluorescence intensity of detected aggregates of three experimental replicates for Additional file 1: Fig. S4.

## Data Availability

All data generated or analyzed during this study are included in this published article, its supplementary information files, and publicly available repositories. All image data sets and source codes for image analysis were deposited in Figshare (https://doi.org/10.6084/m9.figshare.22232875.v1). Data values for the plots in Figs. S3B, S3C, and S4 were shown as Additional files 3, 4 and 5.

## Abbreviations

DmAgo2: *Drosophila melanogaster* Argonaute2
IDR: Intrinsic disordered domain
Nter: N-terminal region of DmAgo2
PAPA: Prion aggregation prediction algorithm
PCA: Principal component analysis
PLAAC: Prion-like amino acid composition
PrLD: Prion-like domain
RISC: RNA-induced silencing complex
SDD-AGE: Semi-denaturing detergent agarose gel electrophoresis
SDS: Sodium dodecyl sulfate
TEM: Transmission electron microscopy
ThT: Thioflavin-T
