# Supplementary material for: N-terminal region of Drosophila melanogaster Argonaute2 forms amyloid-like aggregates
Source: BMC Biol. 2023 Apr 19;21:78. doi: 10.1186/s12915-023-01569-3 (PMC10114355; doi:10.1186/s12915-023-01569-3)
Supplement: Supplementary file 1 — Additional file 1: Fig. S1. PAPA did not predict Nter as PrLD. Fig. S2. Gel electrophoresis analyses of the purified mCherry-Nter monomer. Fig. S3. Time-lapse images of Nter aggregation in the glass chamber. Fig. S4. Time-course ThT fluorescence measurement of Nter in solution. Fig. S5. Representative images of Nter aggregates. Fig. S6. Characteristics of each type of Nter aggregate. Fig. S7. TEM images of Nter aggregates. Fig. S8. Width distribution of fibril-shaped aggregates. Fig. S9. Comprehensive PrLD prediction for Argonaute family proteins. Fig. S10. Arabidopsis thaliana Ago1 forms amyloid-like aggregates. [file 12915_2023_1569_MOESM1_ESM.pdf]

Additional file 1

N-terminal region of Drosophila melanogaster Argonaute2 forms amyloid-like aggregates

Haruka Narita, Tomohiro Shima, Ryo Iizuka and Sotaro Uemura

Department of Biological Sciences, Graduate School of Science, The University of Tokyo, Japan

Contents,

Supplementary Figure S1. PAPA did not predict Nter as PrLD

Supplementary Figure S2. Gel electrophoresis analyses of the purified mCherry-Nter monomer

Supplementary Figure S3. Time-lapse images of Nter aggregation in the glass chamber

Supplementary Figure S4. Time-course ThT fluorescence measurement of Nter in solution

Supplementary Figure S5. Representative images of Nter aggregates

Supplementary Figure S6. Characteristics of each type of Nter aggregate

Supplementary Figure S7. TEM images of Nter aggregates

Supplementary Figure S8. Width distribution of fibril-shaped aggregates

Supplementary Figure S9. Comprehensive PrLD prediction for Argonaute family proteins

Supplementary Figure S10. *Arabidopsis thaliana* Ago1 forms amyloid-like aggregates

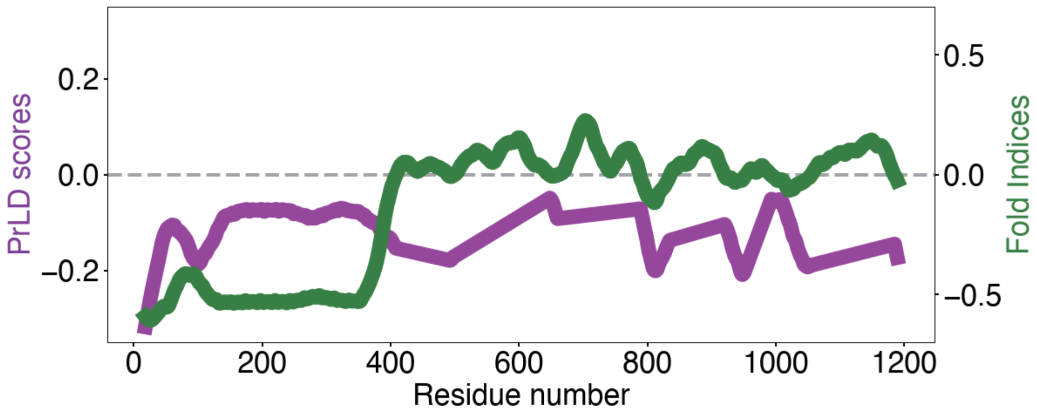

Supplementary Figure S1. PAPA did not predict Nter as PrLD

The expected PrLD scores of DmAgo2 Nter predicted by PAPA. The green line indicates prion formation propensity, and the magenta line indicates fold indices.

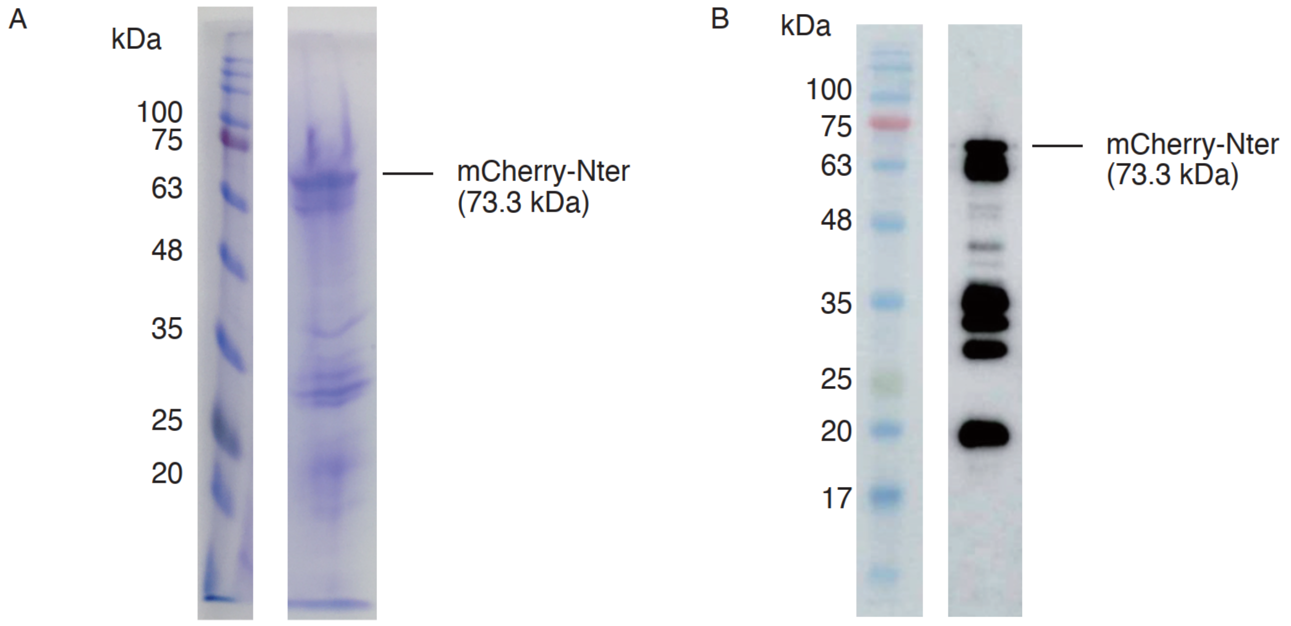

Supplementary Figure S2. Gel electrophoresis analyses of the purified mCherry-Nter monomer
The mCherry-Nter recombinant protein was expressed in *E. coli* and purified through its N-terminal 6xHis-tag. After ultracentrifugation, mCherry-Nter was subjected to SDS-PAGE and stained by Coomassie Brilliant Blue (**A**) or a polyclonal anti-RFP antibody (MBL, PM005) and HRP-labeled IgG detector (**B**, Takara Bio, Western BLoT Rapid Detect v2.0). We confirmed that the solution did not contain oligomers of mCherry-Nter, although there were some small fragments in the solution.
Both images (**A, B**) show additional bands besides the band corresponding to molecular weight of mCherry-Nter (73 kDa). DsRed-derived proteins, including mCherry, are known to be cleaved into N-terminal 10 kDa and C-terminal 20 kDa fragments when boiled in SDS buffer (26). Therefore, the band visible around 63 kDa is likely the C-terminal fragment of mCherry-Nter that was cleaved during SDS-PAGE sample preparation. The 73 and 63 kDa bands account for 60% of the CBB staining signal (**A**), suggesting that the major protein in this sample used in the other experiments is intact mCherry-Nter. The other bands in the CBB-stained gel appeared around 35 kDa (**A**). These ~35 kDa bands were also visible in the Western blot image (**B**), suggesting that these bands contain at least a part of mCherry with N-terminal fragments of Nter. Because the molecular weight of mCherry with linker was 29 kDa, a large portion of these ~35 kDa fragments would consist of mCherry, and only contains several tens of N-terminal residues of Nter. We have tried but failed to isolate the full-length mCherry-Nter from the fragments using size-exclusion chromatography because mCherry-Nter started to aggregate in the column. Therefore, we used this mixture of the full-length and fragments of mCherry-Nter as a purified mCherry-Nter solution in this study.

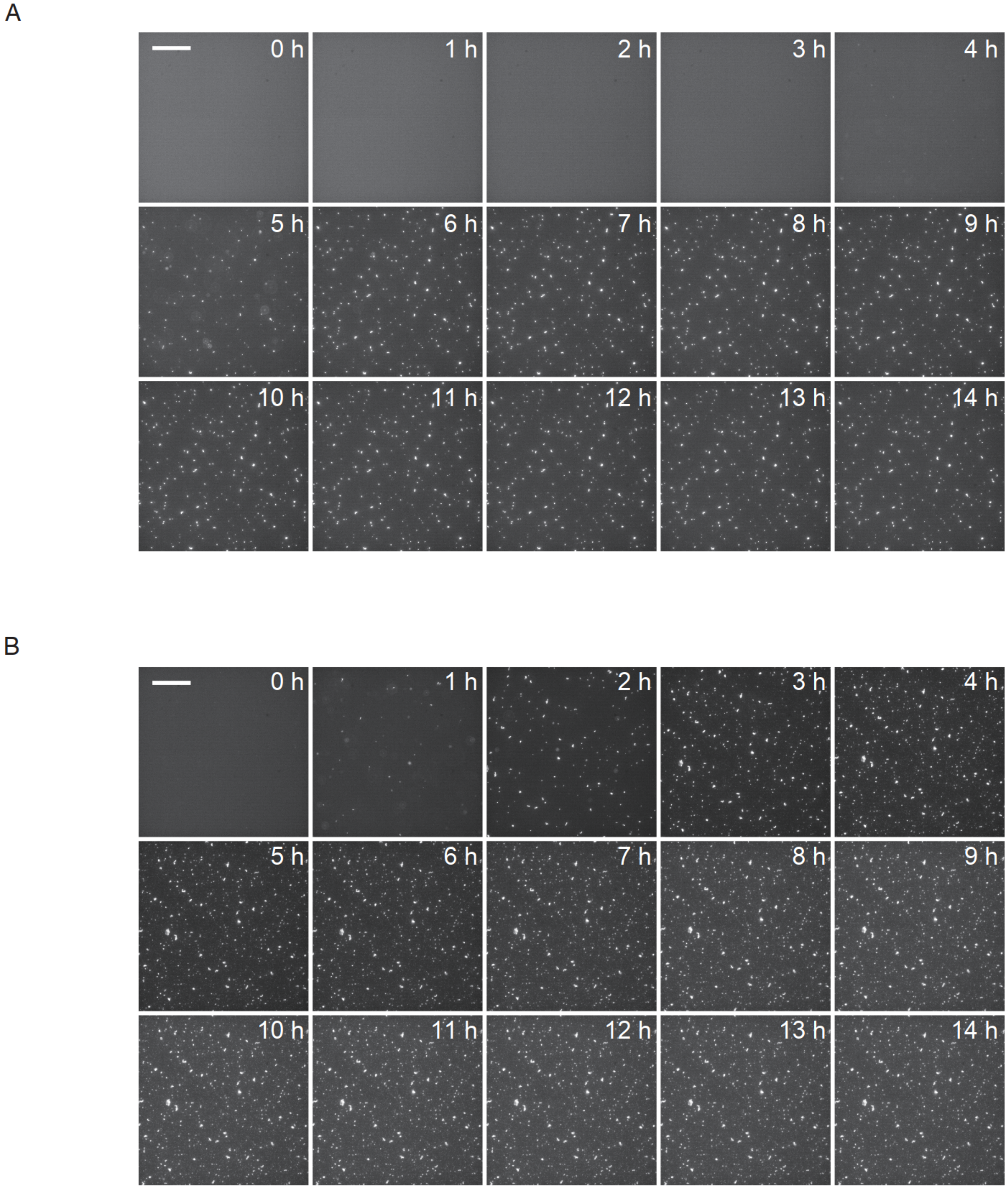

Supplementary Figure S3. Time-lapse images of Nter aggregation in the glass chamber
Fluorescence images of ThT with 5 μM mCherry-Nter in the absence (**A**) and presence (**B**) of seeds. Time interval between images is 1 h. Scale bars, 50 μm.

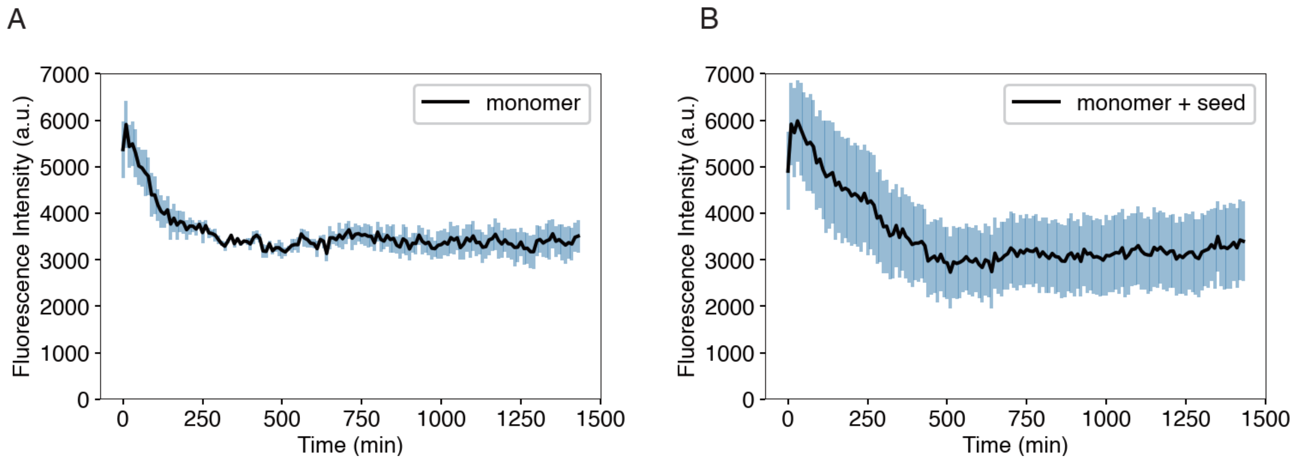

Supplementary Figure S4. Time-course ThT fluorescence measurement of Nter in solution. Time-course of ThT fluorescence intensity of mCherry-Nter solution in the absence (**A**) and presence (**B**) of the aggregate seeds acquired by a microplate reader. The solid black curves and the blue area indicate the mean values and standard deviations of three repetitive experiments (n = 3), respectively.

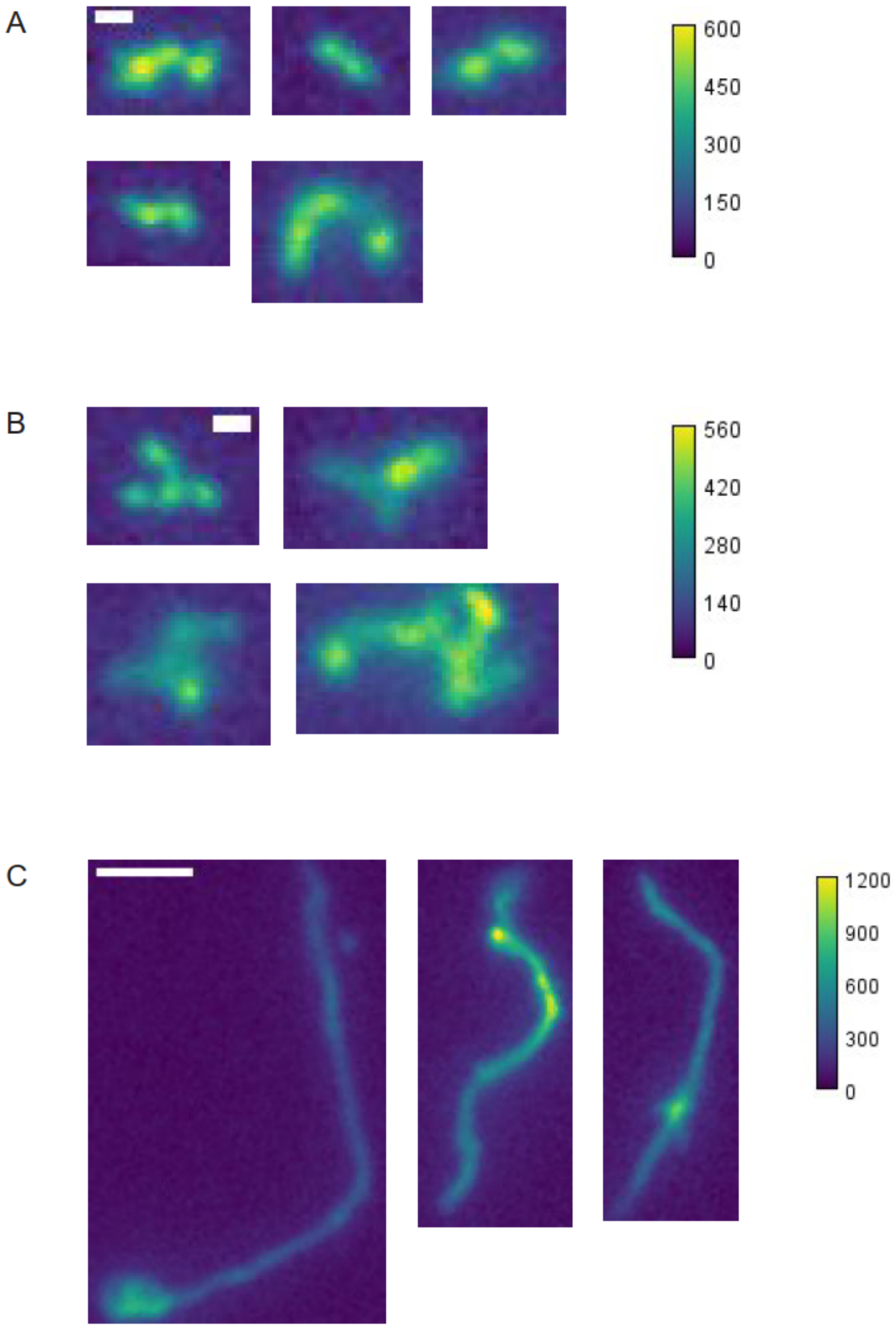

Supplementary Figure S5. Representative images of Nter aggregates

ThT fluorescent images of linear fractal-shaped (**A**), branched fractal-shaped (**B**) and fibril-shaped (**C**) Nter aggregates. The color bar shows the fluorescence intensity (a.u.).

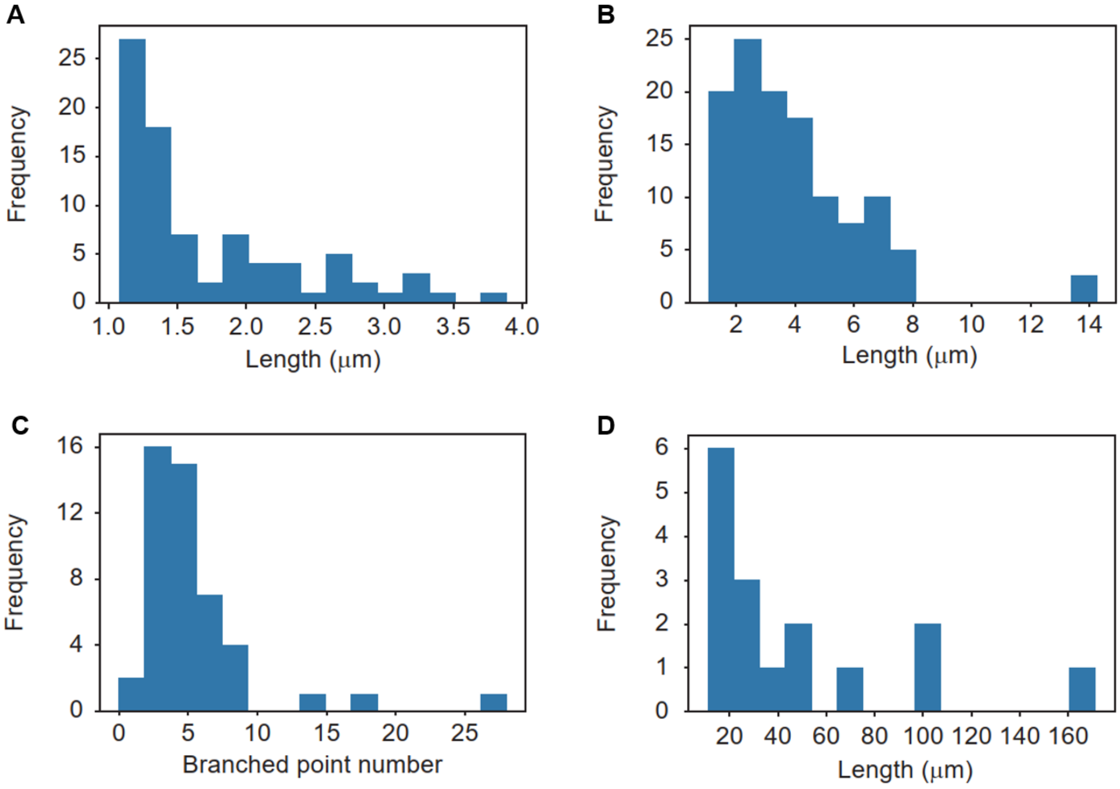

Supplementary Figure S6. Characteristics of each type of Nter aggregate

(A) Length of linear fractal-shaped aggregates.

(B, C) Length (**B**) and number of branches (**C**) of branched fractal-shaped aggregates.

(**D**) Length of fibril-shaped aggregates.

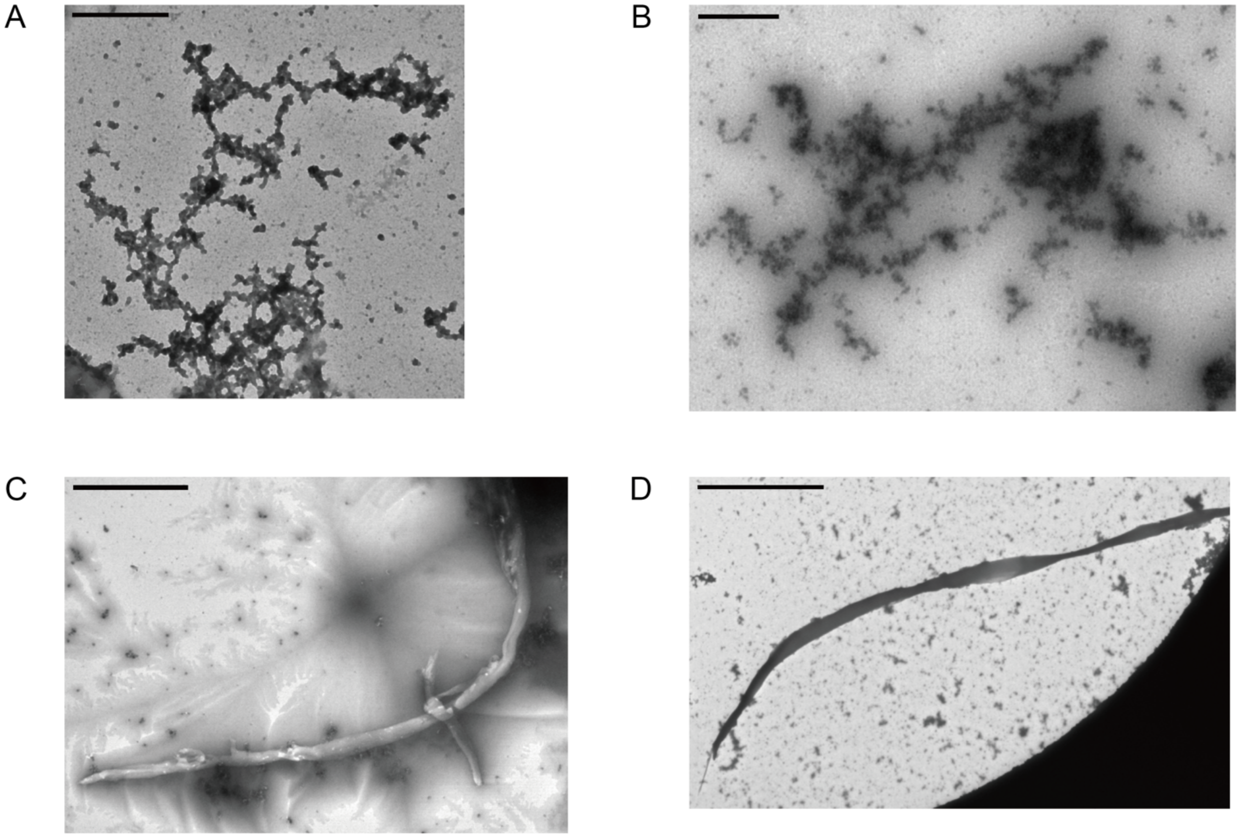

Supplementary Figure S7. TEM images of Nter aggregates

TEM images of fractal-shaped (**A**, **B**) and fibril-shaped aggregates (**C**, **D**). Scale bars represent 500 nm (**A**, **B**), 1 μm (**C**), and 10 μm (**D**).

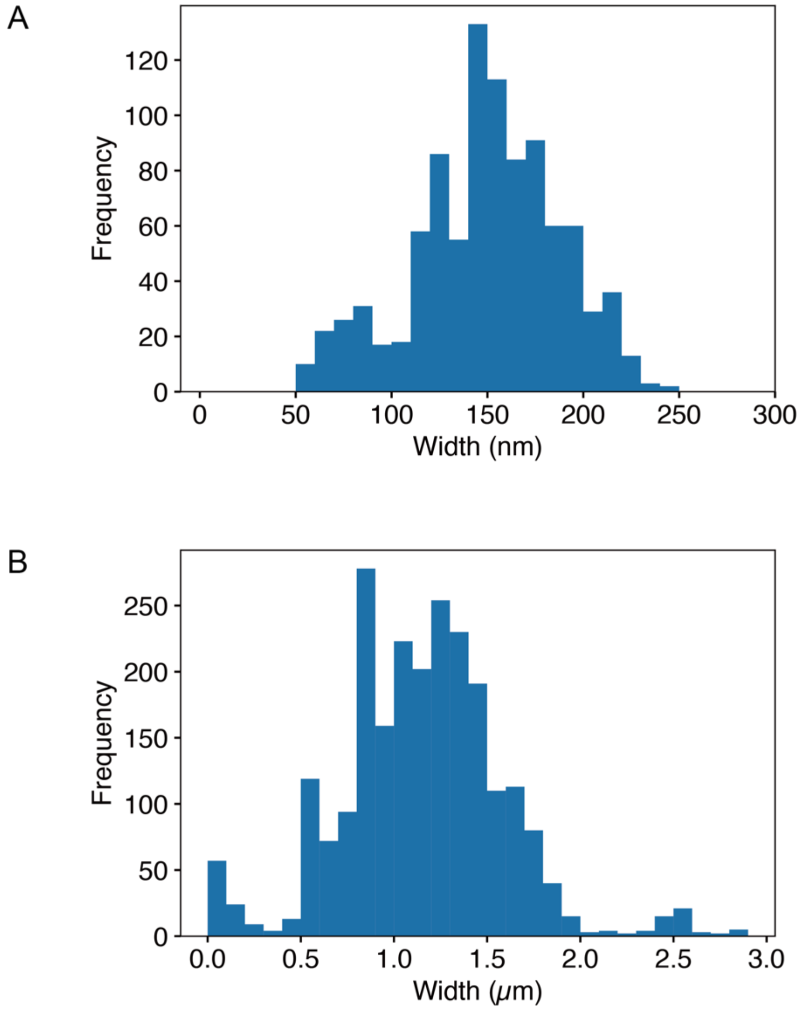

Supplementary Figure S8. Width distribution of fibril-shaped aggregate

Width of fibril-shaped aggregates indicated in Supplementary Figure 7C (**A**) and 7D (**B**).

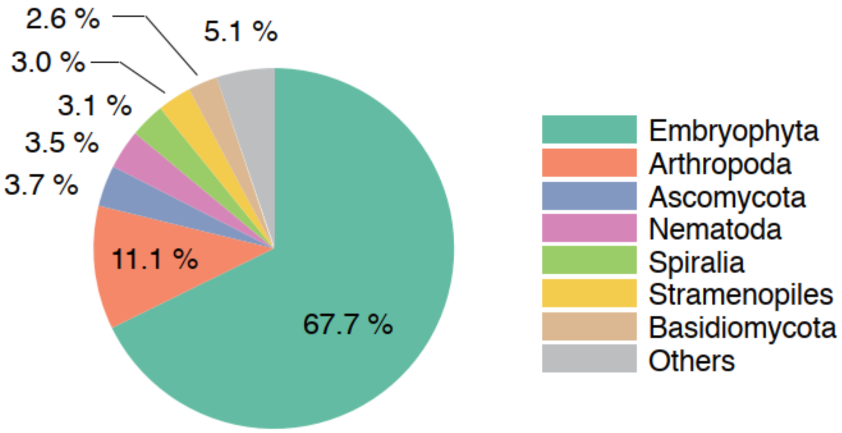

Supplementary Figure S9. Comprehensive PrLD prediction for Argonaute family proteins
Taxonomic domain distribution of the Argonaute family genes predicted to harbor a PrLD. Among 14,239 sequences of full-length Argonaute family proteins in the UniProt database, 1,246 proteins were predicted to harbor a PrLD at their N-terminus by PLAAC. The diversity of taxonomic groups appeared here suggests that the property of having PrLD at the N-terminus of Argonaute proteins is widely conserved in the biological world.

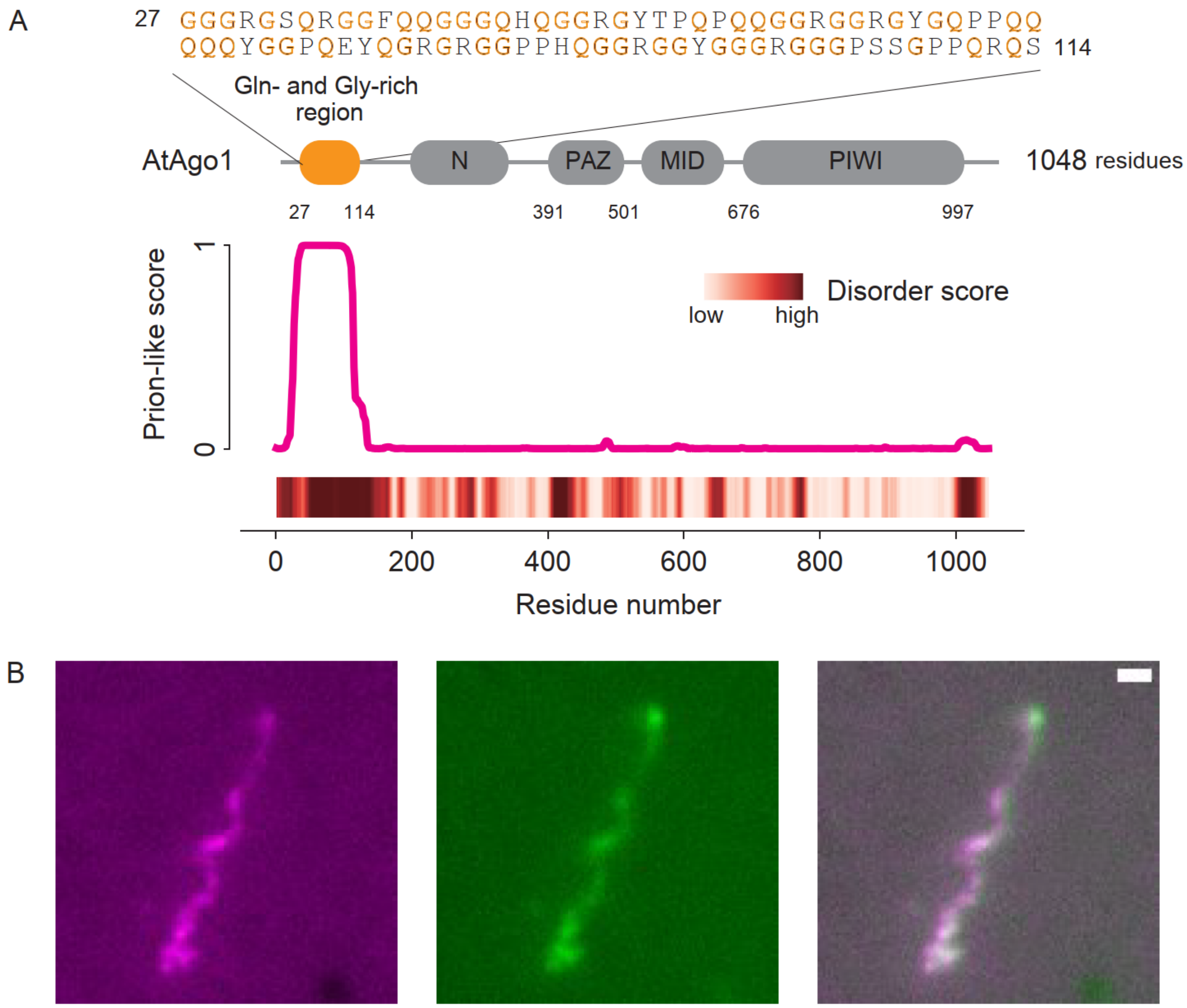

Supplementary Figure S10. ***Arabidopsis thaliana*** **Ago1 forms amyloid-like aggregates**

(A) A schematic diagram (top), plot of the prion-like probability predicted by PLAAC (middle) and a heatmap of the degree of disorder predicted by PONDR analysis (bottom) of *Arabidopsis thaliana* Ago1 (AtAgo1). The PLAAC algorithm showed high prion-like probability in the N-terminal (residues 27–114) of AtAgo1. (**B**) Representative fluorescence images of the aggregated N-terminal region in AtAgo1. The N-terminal region (residues 26–114) was fused with 6xHis and mCherry at its N-terminus, expressed in *E. coli* and purified using Ni-NTA resin, same as DmAgo2. The purified protein formed large aggregates during one day of incubation. Fluorescence signals from mCherry (left, magenta) and ThT (middle, green) showed almost identical images (merged image, right) of the aggregate, suggesting that the N-terminal of AtAgo1 also has the ability to form amyloid-like aggregates. Scale bar represents 1 μm.
