# Supplementary figures and images for: N-terminal region of Drosophila melanogaster Argonaute2 forms amyloid-like aggregates
Source: BMC Biol. 2023 Apr 19;21:78. doi: 10.1186/s12915-023-01569-3 (PMC10114355; doi:10.1186/s12915-023-01569-3)

## Additional file 2

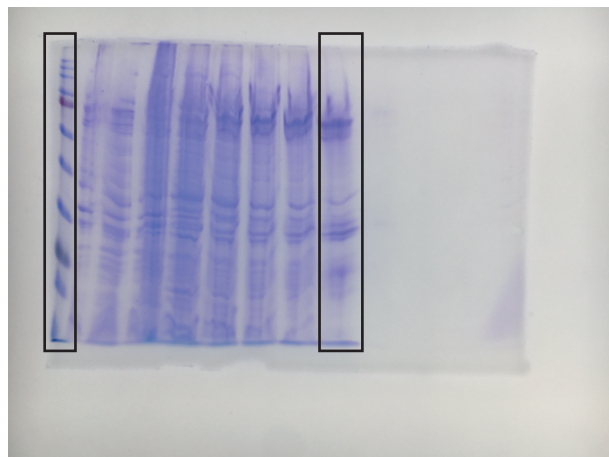

Uncropped Image 1

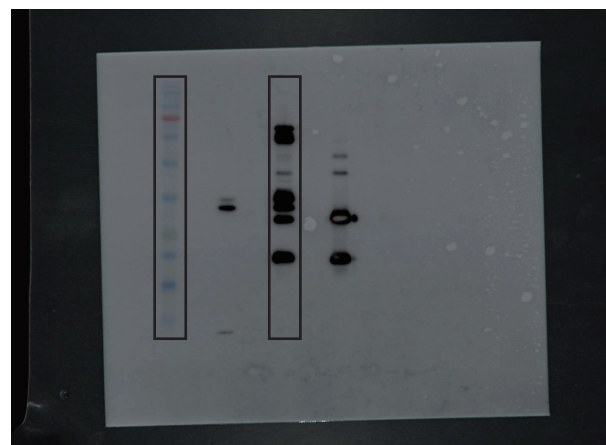

Uncropped Image 2

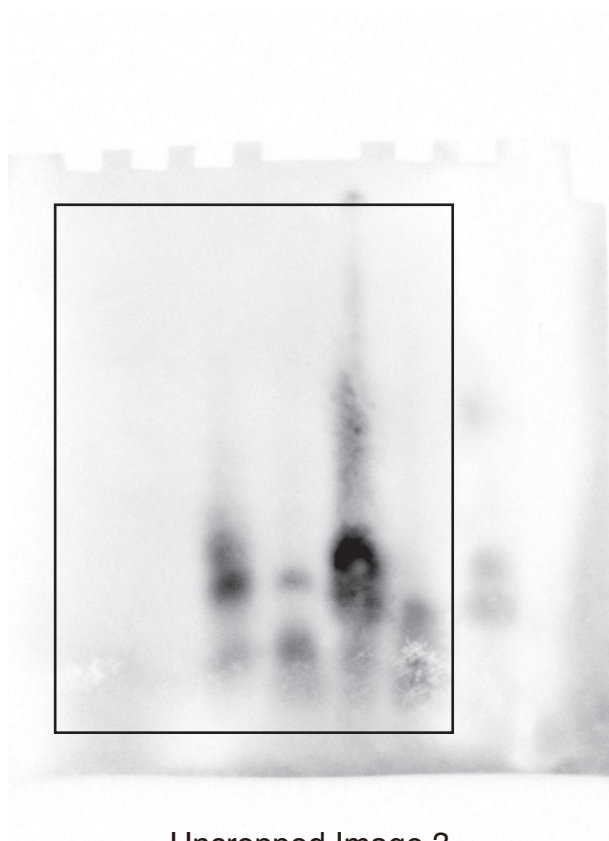

Uncropped Image 3

Supplement: Supplementary file 2 — Additional file 2: Uncropped Image 1. Uncropped gel image used for Additional file 1: Fig. S2A (A). Uncropped Image 2. uncropped blot image used for Additional file 1: Fig. S2B (B). Uncropped Image 3. Uncropped blot image used for Fig. 2. [file 12915_2023_1569_MOESM2_ESM.pdf]
